# Remodeling the Immune Landscape: How Obesity-Induced SASPs Create a Pro-Tumorigenic Niche

**DOI:** 10.1007/s13679-026-00750-3

**Published:** 2026-08-22

**Authors:** Eslam E. Abd El-Fattah, Gary Ngai, Rachael Mooney, Karen S. Aboody

**Affiliations:** Department of Stem Cell Biology and Regenerative Medicine, City of Hope Beckman Research Institute, Duarte, CA USA

**Keywords:** Obesity, Cancer, SASP, Adipose, Inflammation, Tumor microenvironment

## Abstract

**Purpose of Review:**

Obesity is a potent risk factor for cancer, primarily driven by a state of chronic inflammation. A key mechanism linking adipose tissue dysfunction to tumorigenesis is the induction of cellular senescence and the related Senescence-Associated Secretory Phenotype (SASP), which is not clearly defined.

**Recent Findings:**

In obesity, expanding adipose tissue becomes hypoxic and stressed, triggering premature senescence in adipocytes and stromal cells. These senescent cells persistently secrete a robust arsenal of SASP factors, including pro-inflammatory cytokines (e.g., IL-6, IL-1β) and chemokines (e.g., IL-8, MCP-1). This review delineates how obesity-related SASPs create a permissive environment for cancer initiation and progression. Systemically, these SASPs establish a low-grade inflammatory milieu that promotes genomic instability and epithelial cell transformation. Within the tumor microenvironment, SASP components directly stimulate cancer cell survival, proliferation, invasion, and angiogenesis, concurrently through immune modulation. Crucially, obesity-related metabolic hormones regulate this process; hyperleptinemia, hyperinsulinemia, and hyperresistinemia amplify SASPs, while hypoadiponectinemia removes a key inhibitory brake.

**Summary:**

Obesity-induced SASPs act as critical endocrine and paracrine signals that remodel tissue landscapes to be pro-tumorigenic. Targeting the SASPs or the metabolic drivers of senescence presents a promising therapeutic strategy to mitigate the potential oncogenic consequences of obesity.

## Introduction

### Obesity and Cancer

Inflammation originating from dysfunctional adipose tissue in patients with obesity is linked to oncogenesis. The initial event is adipocyte hypertrophy due to positive energy balance, which leads to hypoxia and endoplasmic reticulum stress and ultimately cell death, an active initiating event that drives local inflammation and recruitment of immune cells to adipose tissue [1–4]. The subsequent inflammatory signaling from stressed adipocytes recruits and activates immune cells, thereby establishing a pro-tumorigenic systemic environment [5].

A robust body of epidemiological evidence confirms that obesity significantly elevates the risk of developing various cancers, including those of the breast [6], colorectum [7], endometrium [8], kidney [9], and liver [10]. The association between obesity and aggressive malignancies, such as the two-fold risk of hepatocellular carcinoma (HCC)-related mortality identified by Gupta, Das [11], is critically mediated by a state of chronic inflammation and immune dysregulation originating from dysfunctional adipose tissue. Obesity promotes oncogenesis through multiple interacting mechanisms, including hyperinsulinemia, dyslipidemia, and dysregulation of sex hormones and growth factors. Nevertheless, adipose tissue-driven immune dysregulation encompassing chronic low-grade inflammation, aberrant adipokine secretion, and remodeling of the tumor microenvironment, represents a central and unifying mechanistic axis. This review article will synthesize our current understanding of how obesity remodels the immune landscape to actively promote tumor initiation, progression, and therapy resistance.

It should be noted, however, that while the epidemiological association between obesity and cancer risk is consistently demonstrated across large cohort studies and meta-analyses, the attribution of cancer risk to adipose-driven immunopathology specifically as distinct from co-occurring metabolic perturbations such as hyperinsulinemia, sex hormone dysregulation, and dyslipidemia remains difficult to disentangle in human observational data. Establishing causality and identifying the dominant mechanistic pathway in clinical populations are central challenges in this field, and the mechanistic framework presented in this review draws primarily from preclinical models that may not fully replicate the heterogeneity of human obesity-associated malignancies.

### Obesity and Immune Dysregulation

The local inflammatory milieu of dysfunctional adipose tissue exerts systemic effects, reprogramming the host’s immune profile resulting in an increased percentage of pro-inflammatory (M1) macrophages that secrete cytokines such as TNF-α, and IL-6 [12]. This reprogramming directly impinges upon the tumor microenvironment (TME), establishing an immunosuppressive niche characterized by diminished anti-tumor activity and enhanced pro-tumorigenic processes [13]. It does this by undermining the adaptive immune response metabolic dysregulation of CD8 + T cells. Exposure to a lipid-rich microenvironment drives cytotoxic T lymphocytes (CTLs) into a state of functional exhaustion by disrupting their bioenergetic processes. This impairment in mitochondrial metabolism and ATP generation directly attenuates their cytotoxic potential, leading to ineffective tumor cell clearance. This metabolic insult is frequently accompanied by the upregulation of inhibitory receptors such as programmed death receptor 1 (PD-1) and cytotoxic T lymphocyte antigen 4 (CTLA-4) on tumor-infiltrating CD8 + T cells, pushing them toward a dysfunctional exhausted state. Consequently, their effector functions are attenuated, enabling tumors to evade immune destruction [14].

While this lipid-driven CTL exhaustion model is well supported by in vitro lipid-loading experiments and murine dietary obesity models, direct evidence linking adiposity to CD8^+^ T cell bioenergetic dysfunction within the TME in human obesity-associated cancers remains limited. Whether the degree of lipid accumulation achieved in experimental models accurately reflects the lipid microenvironment of human tumors in individuals with obesity is an important and largely unresolved translational question.

Beyond impairing cytotoxic lymphocytes, obesity actively fosters an immunosuppressive landscape by recruiting and activating cells that antagonize anti-tumor immunity [15]. Systemic inflammatory mediators derived from dysfunctional adipose tissue promote the expansion and trafficking of myeloid-derived suppressor cells (MDSCs) to the TME [16]. Within the TME, these immature myeloid cells potently suppress the activity of both T cells and natural killer (NK) cells, creating a protective barrier around the tumor [17]. This immunomodulatory rewiring has direct clinical ramifications, as emerging evidence indicates that obesity can diminish the efficacy of immune checkpoint inhibitors (e.g., anti-PD-1/PD-L1), although the underlying mechanisms remain an active area of investigation [18]. Consequently, the immunosuppressive architecture of patients with obesity TME, dominated by MDSCs and functionally impaired CTLs, presents a major barrier to the efficacy of immunotherapies that require a pre-existing immune response for optimal activity [19–21].

Circulating myeloid compartment perturbation is a consistent feature of obesity. In a human cohort study, Friedrich, Sommer [22] demonstrated that individuals with obesity exhibit significantly higher proportions of total monocytes, classical monocytes (CM), intermediate monocytes (IM), CD56 + monocytes, and monocytic MDSCs (M-MDSCs) compared to lean controls. These elevations correlate positively with indices of adiposity and metabolic dysfunction including BMI, body fat percentage, waist circumference, triglycerides, C-reactive protein, and HbA1c and inversely with HDL cholesterol, establishing a direct link between metabolic dysregulation and myeloid immune skewing [22]. The functional significance of this MDSC expansion is illustrated in preclinical models: in *ob/ob* mice with ovarian cancer, MDSC accumulation within the tumor microenvironment was associated with elevated IL-6, suggesting that obesity-driven hyperleptinemia and SASP amplification actively recruit and retain immunosuppressive myeloid populations in the TME [23].

Resistance to conventional cancer therapies is further exacerbated by the patients with obesity microenvironment. Systemic inflammation, including persistent IL-6 signaling, alongside adipokine-driven activation of survival pathways (e.g., leptin), allows malignant cells to circumvent apoptotic mechanisms triggered by chemo- and radiotherapy [24–26]. This convergence of pro-survival signals ultimately manifests as treatment failure and worsened survival outcomes [24–27]. A hallmark of adipose tissue inflammation in obesity is a profound shift in macrophage polarization and abundance. Under lean conditions, adipose tissue-resident macrophages exhibit a predominantly M2-polarized, anti-inflammatory state that supports tissue homeostasis and lipid handling [28]. M2-biased macrophages are more prevalent in breast adipose tissue than M1-biased macrophages in all BMI categories [29]. Obesity did not alter the density of M1-biased macrophages, but it did increase M2-biased macrophages. According to Gene Set Enrichment Analysis, breast tissue macrophages from obese women resemble tumor-associated macrophages (TAM) more than those from lean women. In vitro tests revealed that obesity extracellular matrix (ECM) directly increases M2-biased macrophage activities, and these alterations were positively linked with adipose tissue interstitial fibrosis [29]. In prostate cancer model, Obese mice exhibited a higher concentration of CD206⁺ TAMs compared to non-obese mice when analysis was restricted to high-grade tumors [30]. Besides, Reduced serum adiponectin levels play a significant role in driving macrophage polarization in obesity. Macrophages isolated from the peritoneal cavity and stromal vascular fraction of adiponectin-deficient mice display an M1 phenotype, characterized by elevated expression of *Tnfα*, *Il6*, and *Ccl2*, alongside reduced expression of M2-associated genes such as *Il10* and arginase-1 [31].

With obesity, however, a substantial infiltration of macrophages occurs, coupled with a phenotypic switch toward a pro-inflammatory M1, or “classically activated,” state. These M1 macrophages become a primary source of inflammatory cytokines that perpetuate local and systemic dysfunction [32]. Beyond classical M1 polarization, obesity is also associated with the emergence of a phenotypically distinct population of “metabolically activated” macrophages (MMe). Unlike M1 macrophages, which are driven by immunological signals such as LPS and IFN-γ, MMe macrophages are activated by the metabolic stimuli that characterize obesity — including elevated glucose, insulin, and saturated fatty acids such as palmitate [33, 34]. Despite their distinct activation mechanism, MMe macrophages are equally capable of secreting pro-inflammatory cytokines and therefore represent an independent, metabolically driven source of SASP amplification in patients with obesity adipose tissue. The co-existence of classical M1 and MMe macrophages in obesity thus underscores that the simple M1/M2 binary is an oversimplification; the true inflammatory landscape of patients with obesity adipose tissue is considerably more heterogeneous, with multiple macrophage activation states converging to sustain the chronic pro-tumorigenic environment [35].

A mechanistically distinct but underappreciated driver of adipose tissue inflammation in obesity is adipocyte cell death itself. As adipocytes undergo progressive hypertrophy beyond their oxygen diffusion capacity, they become susceptible to necrosis, apoptosis, and pyroptosis — collectively releasing intracellular contents including lipid droplets, free fatty acids (FFAs), and damage-associated molecular patterns (DAMPs) such as HMGB1 and mitochondrial DNA into the extracellular space [36]. These DAMPs activate pattern-recognition receptors notably TLR2 and TLR4, triggering NF-κB-driven pro-inflammatory signaling and initiating the recruitment of circulating monocytes to sites of adipocyte death [37, 38].

Adipocyte death is thus not merely a passive consequence of adipose tissue stress, but an active initiating event that shapes the subsequent inflammatory response. Furthermore, the loss of viable secretory adipocytes directly disrupts the adipokine landscape: adiponectin production which requires intact adipocyte function falls precipitously, while the inflammatory milieu surrounding dying cells paradoxically upregulates leptin, resistin, and pro-inflammatory cytokines (TNF-α, IL-6, MCP-1) in surviving hypertrophied adipocytes, tilting the adipokine balance decisively toward a pro-tumorigenic secretory profile [39]. The progressive replacement of functional adipocytes by lipid-engorged, dead, or senescent cells thereby reduces the lipid-buffering capacity of adipose tissue, promotes ectopic lipid deposition, deepens insulin resistance, and amplifies the chronic inflammatory state [40, 41] creating a self-sustaining cycle of adipose tissue dysfunction that may contribute to obesity-associated oncogenesis.

The accumulation of dead adipocytes triggers the clustering of recruited M1-polarized macrophages into crown-like structures (CLSs), which form specifically to phagocytose the lipid contents of necrotic cells and clear cellular debris [42]. These CLSs mark a self-sustaining inflammatory focus: the macrophages within them secrete TNF-α [43]. This recruitment-and-polarization feed-forward loop sustains chronic adipose tissue inflammation and, critically, further suppresses adiponectin secretion from surrounding adipocytes through paracrine TNF-α signaling, thereby locking the adipokine imbalance created by initial adipocyte death into a stable, pro-tumorigenic state [44, 45].

Adipose tissue functions as a dynamic endocrine organ, secreting a repertoire of bioactive signaling molecules termed adipokines. In obesity, the secretion profile of these adipokines becomes profoundly dysregulated, shifting the balance toward a milieu that promotes oncogenic signaling [46]. The adipokine landscape is broad, encompassing classical molecules such as leptin, adiponectin, and resistin alongside emerging mediators including visfatin, chemerin, FGF21, and omentin-1, all of which have been implicated to varying degrees in obesity-associated tumor biology [47–49].

Leptin exerts pleiotropic effects in obesity-associated cancer by signaling through the leptin receptor (Ob-R) [50]. Leptin acts as a potent mitogen on malignant cells [51], driving tumor progression by promoting proliferation [52], inhibiting apoptosis [53], stimulating angiogenesis [54], and enhancing metastatic potential [55]. Concurrently, leptin signaling on immune cells skews T helper cell differentiation toward pro-inflammatory Th1 function and Th17 phenotypes [56].

In contrast to leptin, adiponectin is an adipokine with potent anti-inflammatory and insulin-sensitizing properties, whose circulating levels are inversely correlated with adipose mass. Adiponectin exerts direct anti-tumor effects by inhibiting cancer cell proliferation and promoting apoptosis. Consequently, the obesity-associated decline in adiponectin levels effectively removes a critical endogenous brake on tumorigenesis [57]. While not classical adipokines, pro-inflammatory cytokines like TNF-α and IL-6, primarily secreted by adipose tissue-infiltrating macrophages, are central mediators of obesity-associated inflammation [58]. IL-6 is particularly pivotal, activating the STAT3 oncogenic signaling pathway in tumor cells to foster survival, proliferation, and invasion, thereby critically bridging chronic inflammation to malignant progression [59].

## Effect of Obesity on Senescence

The pathophysiological impact of obesity extends to the acceleration of biological aging, primarily through the induction of cellular senescence. This is orchestrated by metaflammation, a state of chronic, adipose tissue-driven inflammation that systemically disrupts tissue function. A central mechanism is the cellular stress response to persistent nutrient excess, which activates pathways leading to premature senescence [60]. Expanding adipose tissue, particularly visceral fat, becomes dysfunctional, failing to sequester lipids safely and instead releasing a flood of pro-inflammatory adipokines (like leptin and resistin) and cytokines (such as TNF-α and IL-6) into the circulation [61–64]. This chronic systemic inflammation constitutes a potential component of SASPs, essentially broadcasting an aging signal throughout the body. The nutrient surplus characteristic of obesity may induce mitochondrial dysfunction, especially in highly metabolic tissues such as skeletal muscle, liver, and pancreatic β-cells [65–67]. This dysfunction elevates reactive oxygen species (ROS) generation, which surpasses antioxidant capacity and creates a state of oxidative stress. The resulting oxidative damage to macromolecules particularly DNA serves as a primary trigger for cellular senescence by activating the canonical p53/p21 and p16/Rb tumor suppressor pathways, which initiate a stable cell cycle arrest [68].

This ROS-to-senescence mechanistic chain is well established in cell culture models and murine dietary obesity paradigms; however, it is important to note that most direct evidence derives from preclinical systems. Cross-sectional human studies have documented positive associations between BMI and markers of oxidative stress and senescence burden in adipose tissue, but these are correlational and do not establish that ROS accumulation in obesity is the primary driver of senescence onset in vivo. The degree of ROS generation required to trigger p53/p21 pathway activation in human adipocytes under physiological obesity conditions rather than in lipid-overloaded cell lines, remains to be directly quantified.

In addition to fostering inflammation and oxidative stress, obesity disrupts core nutrient-sensing pathways that maintain cellular homeostasis. Chronic hyperglycemia and hyperinsulinemia, hallmarks of obesity-related insulin resistance [69], lead to dysregulation of the IGF-1/AKT/mTOR axis, a central regulator of growth and aging [70, 71]. The consequent hyperactivation of mTOR, a characteristic response to nutrient surplus, suppresses autophagy, a critical quality control mechanism for clearing damaged cellular components [72]. Impaired autophagy accelerates senescence by allowing damage to accumulate [73], while concurrent metabolic stresses disrupt telomere integrity, as evidenced by shorter telomeres in individuals with high body mass index (BMI) [74]. The resulting accumulation of senescent cells acts as a persistent source of inflammatory and pro-senescent signals, fostering a vicious cycle that amplifies aging systemically [40]. The pathological consequences of obesity-driven senescence are multisystemic, underpinning common age-related diseases such as atherosclerosis, osteoarthritis and metabolic dysfunction-associated steatohepatitis (MASH). By synergistically disrupting cellular integrity, obesity functions as a potent accelerator of the aging process [75]. The demonstration that senescent features are mitigable by reducing adiposity underscores the profound therapeutic potential of lifestyle interventions for promoting health span [76].

Beyond cellular senescence, the chronic inflammatory milieu of patients with obesity adipose tissue activates multiple additional programmed inflammatory cell death pathways in adipocytes. Pyroptosis, a caspase-1-dependent, proinflammatory form of cell death mediated by gasdermin D pore formation is now recognized as a predominant mode of adipocyte death in obesity, as adipocytes under metabolic stress display ultrastructural features consistent with this pathway and release the inflammasome-processed cytokines IL-1β and IL-18 directly into the tissue environment [36].

A third pathway, necroptosis executed via the RIPK1/RIPK3/MLKL axis, drives programmed cellular necrosis that releases DAMPs including HMGB1 and mitochondrial DNA, which in turn activate TLR4 and the NLRP3 inflammasome on macrophages, fueling CLS formation and macrophage recruitment [77]. These three pathways, pyroptosis, apoptosis, and necroptosis, are not mutually exclusive; they can be activated simultaneously within the same inflammatory context, a convergent phenomenon collectively termed PANoptosis or inflammatory cell death [78]. Critically, PANoptosis and cellular senescence share upstream triggers, including ROS accumulation, mitochondrial dysfunction, and the nutrient excess characteristic of obesity [79–84], yet produce distinct but complementary proinflammatory outputs: senescent adipocytes survive and secrete SASP mediators [85], while PANoptotic cells die acutely and release a burst of IL-1β, IL-18, and DAMPs [86], as compared with other models, that further prime macrophages and amplify the pro-tumorigenic inflammatory niche [87, 88]. Together, these parallel cell fate mechanisms render patients with obesity adipose tissue a self-sustaining source of inflammatory signals that collectively could drive SASP amplification, immune dysregulation, and ultimately oncogenesis.

## Effect of SASPs on Cancer Progression

### Effect of IL-6 on Cancer Progression

Interleukin-6 (IL-6) signaling exerts a net pro-tumorigenic effect on many cancers, where this pleiotropic cytokine contributes to disease pathogenesis by facilitating tumor development, progression, and aggressive clinical behavior [89]. IL-6 exerts its effects primarily via the JAK/STAT3 axis. Following receptor engagement, JAK-mediated phosphorylation activates STAT3, allowing it to translocate to the nucleus and transcribe a pro-tumorigenic program encompassing cell cycle progression, apoptotic resistance, invasion, and blood vessel formation which is a key process in hepatocarcinogenesis [90, 91]. High levels of IL-6 in the TME, largely derived from TAMs, contribute to immunosuppression while simultaneously activating a pro-tumorigenic feedback loop via the STAT3 pathway. This signaling axis drives malignant progression by increasing cancer stem cell properties and invasive capacity [92]. The clinical significance of this pathway is underscored by the association between high circulating IL-6 levels and adverse outcomes, including early recurrence, treatment resistance, and poor survival, positioning IL-6/STAT3 signaling as a promising target for novel therapies [93].

The pro-tumorigenic role of IL-6 via JAK/STAT3 is one of the best-supported mechanisms in cancer biology, with consistent evidence across multiple cancer types, in vitro systems, and animal models. Importantly, elevated circulating IL-6 has been associated with poor prognosis in human clinical studies across breast [94], colorectal [95–97], and hematological malignancies [98–100], lending this axis a degree of translational validity that not all SASP factors yet possess. A notable area of controversy, however, is the finding that IL-6 also plays roles in anti-tumor immune activation particularly in the early stages of tumor surveillance meaning that systemic IL-6 blockade carries the theoretical risk of impairing protective immunity [101, 102]. This dual role suggests that therapeutic targeting of IL-6 signaling will require careful patient stratification and context-specific dosing strategies.

### Effect of IL-8 on Cancer Progression

In cancer, elevated levels of the chemokine IL-8 (CXCL8), a key driver of tumor invasion and metastasis, are strongly associated with aggressive clinicopathological features, such as advanced tumor stage, vascular invasion, and early recurrence, culminating in poor patient survival [103–105]. The role of IL-8 extends to shaping an immunosuppressive TME, and its elevated levels are associated with innate resistance to immune-based and systemic therapies. This dual function provides a compelling rationale for developing IL-8/CXCR1/2 axis inhibitors to reverse immunosuppression and improve therapeutic outcomes in cancer [106–110].

The prognostic and mechanistic evidence for IL-8 in cancer is growing but is currently less uniformly established across cancer types than the IL-6/STAT3 axis. Much of the mechanistic data for IL-8-driven invasion, angiogenesis, and immunosuppression derives from in vitro cell line experiments or xenograft models; large-scale human clinical data validating IL-8 as an independent driver, rather than a correlate, of aggressive biology in obesity-associated cancers specifically are sparse. IL-8/CXCR1/2 inhibitors remain in early-phase clinical development, and their therapeutic utility in the obesity-cancer context is currently speculative rather than evidence-based.

### Effect of MCP-1 on Cancer Progression

Monocyte chemoattractant protein-1 (MCP-1/CCL2) exerts a profound pro-tumorigenic effect in HCC, primarily by orchestrating an immunosuppressive TME. MCP-1 is upregulated in both HCC tissue and patient serum, where it functions to recruit CCR2-expressing monocytes and macrophages into the liver, a critical step in tumor progression [111]. Upon infiltration, these CCR2 + monocytes differentiate into TAMs, predominantly adopting an M2-polarized, pro-tumorigenic phenotype [112–114]. These M2 TAMs facilitate cancer progression by secreting a repertoire of growth factors and immunosuppressive cytokines that create a permissive tumor niche [115, 116]. Independent of its role in recruitment, the CCL2/CCR2 axis directly enhances HCC malignancy by activating oncogenic pathways such as Hedgehog (Hh) and TGF-β. This direct signaling promotes key aggressive traits, including cancer cell migration, invasion, angiogenesis, epithelial-mesenchymal transition (EMT), and ultimately, metastatic dissemination and post-surgical recurrence [117]. During obesity, adipocytes in both human and murine breast tissues recruit and stimulate macrophages via a previously unidentified CCL2/IL-1β/CXCL12 signaling pathway [118]. Postmenopausal obesity may enhance CCL2 production in breast adipose tissue, thereby intensifying menopause-associated inflammation and further promoting local aromatase activity and estrogen production, which collectively increase breast cancer risk [119]. In colorectal cancer, adipocytes from obese and CRC subjects were found to release higher amounts of pro-inflammatory and immunoregulatory cytokines/chemokines (IL-6, CXCL8, CCL2, IL-10) with respect to lean healthy subjects [120]. In ovarian cancer model, MCP‑1 secreted by omental adipocytes binds to its receptor CCR2 on ovarian cancer cells, promoting cell migration and omental metastasis through activation of the PI3K/AKT/mTOR pathway and downstream mediators HIF‑1α and VEGF‑A in cell lines, xenograft, and transgenic mouse models. In vivo, neutralization of MCP‑1 significantly reduced tumor burden and improved survival. Overall, the MCP‑1/CCR2 axis between adipocytes and cancer cells plays a key role in facilitating peritoneal metastasis in ovarian cancer [121].

Given that elevated MCP-1 is an independent predictor of poor survival and treatment failure after TACE, targeting the CCL2/CCR2 axis represents a rational and compelling therapeutic strategy for improving outcomes in HCC [122]. While MCP-1’s role as a macrophage recruiter is a broadly conserved biological function, the specific downstream consequences of CCL2/CCR2 axis activation, including TAM polarization states and their pro-tumorigenic outputs — are known to be cancer-type and microenvironment-specific. The degree to which MCP-1-driven immunosuppression in HCC generalized to other obesity-associated cancers such as endometrial, renal, or breast cancer has not been systematically studied, and extrapolation should be made with caution. Furthermore, clinical trials targeting CCL2/CCR2 have yielded modest results in several tumor types, suggesting that MCP-1 is a necessary but not sufficient driver of the immunosuppressive TME.

### Effect of IL-1β on Cancer Progression

**Fig. 1:**
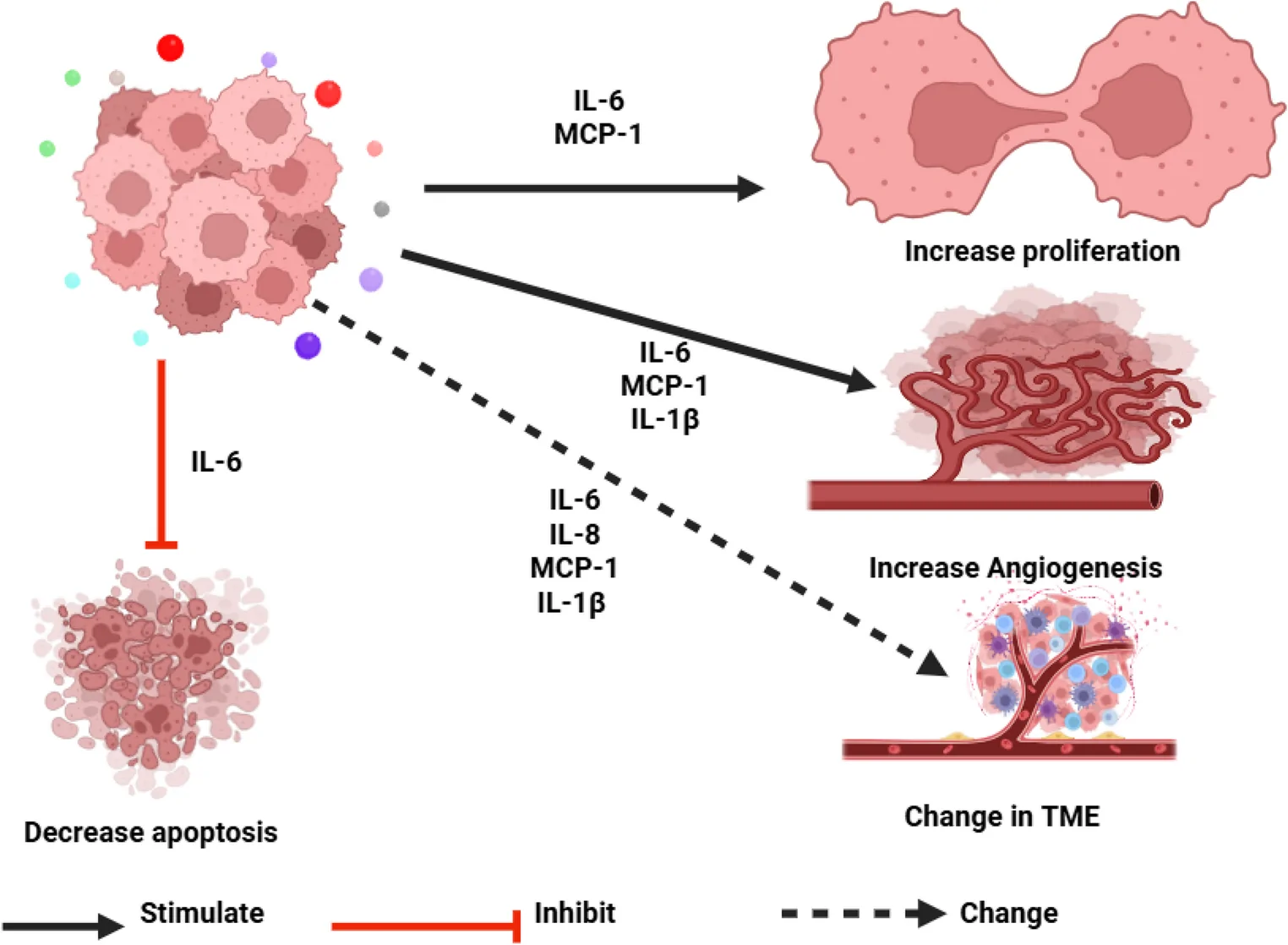
Effects of SASPs secretion on Cancer. Schematic illustration depicting the effects of SAPs on tumor progression and the tumor microenvironment (TME). Adipocyte-derived factors, including IL‑6 and MCP‑1, promote tumor cell proliferation and contribute to enhanced angiogenesis, with additional involvement of IL‑1β and IL‑8. IL‑6 signaling also inhibits apoptosis in tumor cells. Collectively, these cytokine-mediated interactions drive remodeling of the TME, favoring tumor growth and vascularization. Solid arrows indicate stimulation, red lines indicate inhibition, and dashed arrows represent broader changes in the TME. IL-6: Interleukin 6, IL-8: Interleukin 8, IL-1β: Interleukin-1 beta, MCP-1: Monocyte Chemoattractant Protein-1, SASPs: Senescence associated secretory phenotype

Interleukin-1β (IL-1β) is a master pro-inflammatory cytokine that serves as a critical mechanistic link between chronic inflammation and cancer, playing a complex but net pro-tumorigenic role in tumor development and progression [123]. Elevated serum levels of IL-1β, particularly in patients with chronic hepatitis, are significantly associated with an increased risk of cancer development and poorer prognosis [124, 125]. Within TME, IL-1β is predominantly secreted by activated immune cells, notably TAMs, and mediates its effects by signaling through the IL-1R1 receptor on both malignant and stromal cells [126]. IL-1β fosters cancer progression by directly promoting tumor cell invasion [127], stimulating angiogenesis [128], and subverting anti-tumor immunity through PD-L1 induction and MDSC accumulation [129, 130]. Moreover, a key pathogenic feature is the synergistic loop between IL-1β/NF-κB signaling and hypoxia (HIF-1α), which perpetuates a chronic inflammatory state that drives the most aggressive aspects of HCC [131]. Effects of SASPs are illustrated in Fig. 1.

Among the four SASP factors reviewed in this section, IL-1β has the strongest translational evidence for a causal role in human oncogenesis. The CANTOS trial, a large randomized controlled trial of IL-1β blockade with canakinumab, demonstrated a significant and dose-dependent reduction in lung cancer incidence and cancer-related mortality in a metabolically inflamed human population, providing clinical proof-of-concept for the IL-1β-cancer mechanistic axis (NCT01327846). This distinguishes IL-1β from IL-8 and MCP-1, where the evidence remains predominantly preclinical. However, the IL-1β/cancer relationship is not straightforward: IL-1β also contributes to anti-tumor immune activation in certain contexts, and broad systemic blockade in CANTOS was associated with increased fatal infection risk, highlighting the therapeutic complexity of targeting a cytokine with both pro- and anti-tumorigenic roles depending on the immunological context.

## Effect of Obesity Associated with Metabolic Changes on SASPs

Adipose tissue secretes a diverse and expanding repertoire of bioactive mediators — collectively termed adipokines — that extend well beyond the classical molecules discussed here. Adipokines including visfatin (NAMPT), chemerin, FGF21, omentin-1, and apelin have all been implicated in tumor development, progression, and therapeutic resistance, and the full scope of adipose-tumor crosstalk has been comprehensively reviewed elsewhere [47, 48]. The present review focuses specifically on leptin, adiponectin, resistin, and insulin as the four adipokines/metabolic hormones with the most extensively characterized mechanistic links to SASP amplification and obesity-associated oncogenesis. These four were selected on the basis of: (1) the strength and reproducibility of the evidence connecting them to the four principal SASP cytokines reviewed in Sect. Effect of SASPs on Cancer Progression (IL-6, IL-8, MCP-1, and IL-1β); (2) the availability of mechanistic data from both in vitro and in vivo models; and (3) their direct clinical relevance as measurable circulating biomarkers in individuals with obesity. While insulin is not a classical adipokine, its chronic elevation in obesity-associated insulin resistance makes it an obligate component of the metabolic hormonal milieu that drives SASP production, justifying its inclusion within this section.

### Effect of Leptin on SASPs

**Fig. 2:**
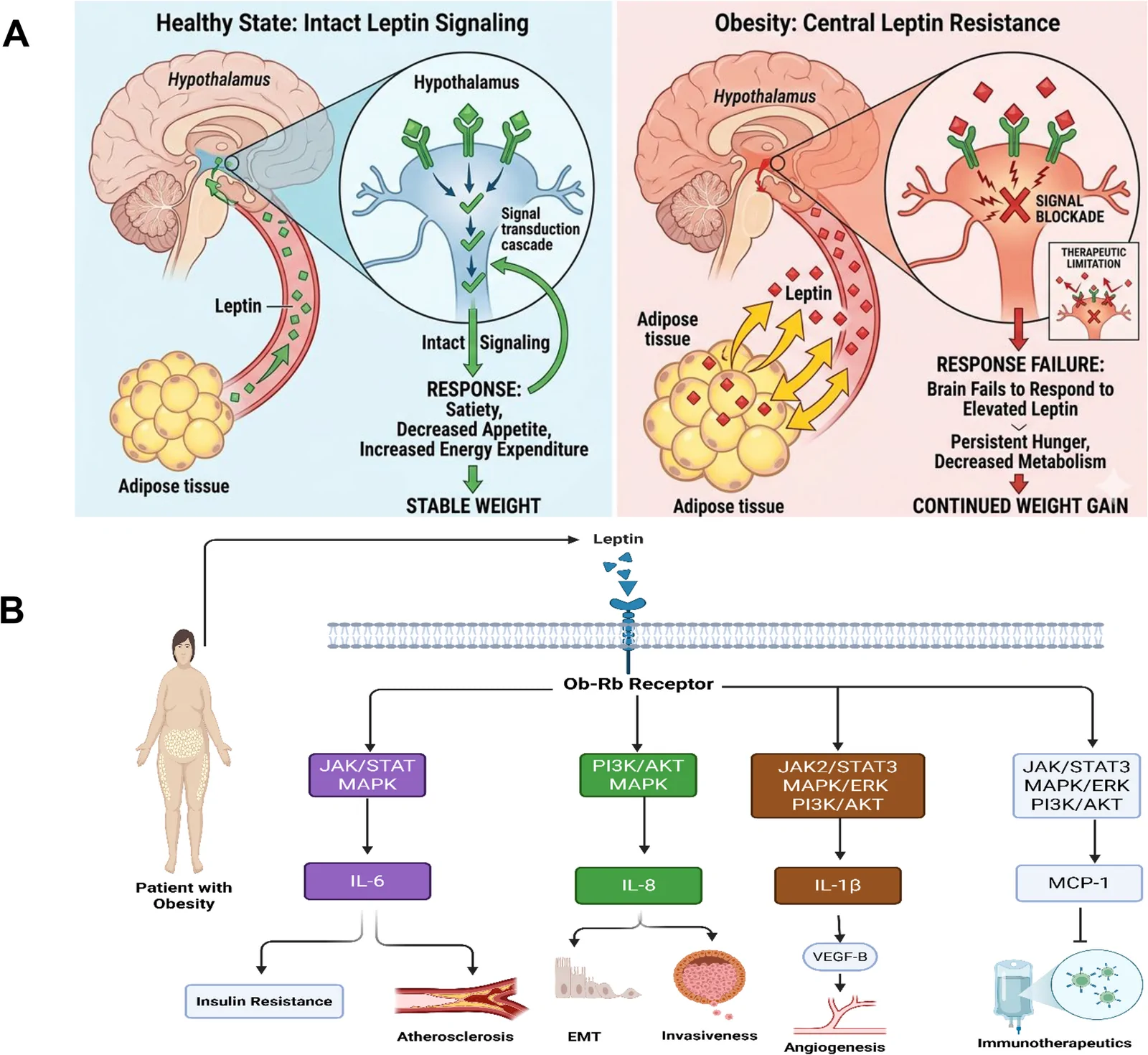
(**A**) Leptin signaling in physiological and obese states. In the healthy state, adipose tissue secretes leptin, which travels through circulation to the hypothalamus, where it binds to leptin receptors on target neurons and activates downstream signal transduction pathways. This intact signaling cascade promotes satiety, suppresses appetite, increases energy expenditure, and maintains stable body weight. In contrast, during obesity, enlarged adipose tissue produces elevated levels of circulating leptin; however, central leptin resistance impairs hypothalamic signaling. Despite receptor engagement, intracellular signal transduction is attenuated or blocked, resulting in a failure to elicit satiety responses. Consequently, appetite remains elevated, energy expenditure is reduced, and continued weight gain occurs. The diagram also highlights therapeutic limitations associated with this signaling blockade (The blue for healthy state and the red half for the obese state). (**B**) Schematic representation illustrating leptin-mediated signaling pathways in obesity and their downstream effects on inflammation, metabolism, and tumor-related processes. In obese individuals, elevated leptin levels activate the Ob‑Rb receptor, triggering multiple signaling cascades including JAK/STAT, MAPK, and PI3K/AKT pathways. These pathways promote the production of IL‑6, IL‑8, IL‑1β, and MCP‑1. IL‑6 contributes to insulin resistance and atherosclerosis, while IL‑8 enhances epithelial–mesenchymal transition (EMT) and tumor cell invasiveness. IL‑1β promotes angiogenesis via VEGF‑B, and MCP‑1 is associated with immune modulation and responses to immunotherapy. Collectively, these signaling events highlight the role of leptin in linking obesity to inflammation, metabolic dysfunction, and cancer progression. Akt: Protein Kinase B, EMT: Epithelial-Mesenchymal Transition, ERK: Extracellular signal-Regulated Kinase, IL-1β: Interleukin-1 beta, IL-6: Interleukin 6, IL-8: Interleukin 8, JAK: Janus kinase, MAPK: Mitogen-Activated Protein Kinase, MCP-1: Monocyte Chemoattractant Protein-1, PI3K: Phosphoinositide 3-kinase, STAT: Signal Transducer and Activator of Transcription, VEGF-B: Vascular Endothelial Growth Factor B

A hallmark of obesity is hyperleptinemia, which occurs due to a breakdown in the hormone’s feedback loop [132]. While leptin normally signals energy sufficiency to the hypothalamus, central resistance in obesity blunts this signal [133]. Consequently, the brain fails to respond to elevated leptin, disrupting energy homeostasis and promoting continued weight gain [134]. Thus, the primary aberration is impaired leptin signal transduction in the brain, rather than a lack of hormone. This inherent resistance fundamentally limits the therapeutic potential of exogenous leptin administration, necessitating alternative strategies to overcome the signaling blockade. Leptin signaling in physiological and obese states is illustrated in Fig. 2A.

An important caveat to the mechanistic framework presented in this section is that most studies linking hyperleptinemia to SASP amplification have been conducted in cell lines or rodent models of diet-induced obesity. Whether leptin resistance, the dominant state in clinical human obesity, preserves or attenuates leptin’s pro-SASP signaling effects in peripheral tissues (as distinct from the brain) remains incompletely resolved. If peripheral leptin resistance parallels central resistance, the functional contribution of hyperleptinemia to SASP-driven oncogenesis in humans may be more complex and attenuated than preclinical models suggest. Effects of leptin on SASPs are illustrated in Fig. 2B.

#### Effect of Leptin on IL-6

The adipokine leptin contributes to chronic, low-grade inflammation, an effect strongly associated with its positive correlation with the pro-inflammatory cytokine IL-6. This relationship between elevated circulating leptin and IL-6 levels is consistently observed in both human studies and animal models of obesity and metabolic syndrome [135]. The mechanistic link involves leptin signaling through its long-form receptor (Ob-R), which directly stimulates IL-6 synthesis and release from a range of cell types, including monocytes, macrophages, T cells, adipocytes, and vascular smooth muscle cells (VSMCs) [136–138]. This process is primarily mediated by the activation of intracellular JAK/STAT and MAPK signaling cascades [139]. Notably, leptin-driven JAK2/STAT3 activation in VSMCs promotes vascular smooth muscle cell proliferation and a pro-inflammatory vascular phenotype, providing a direct cellular mechanism by which hyperleptinemia drives vascular pathology [140].

Consequently, hyperleptinemia functions as an endogenous driver of a pro-inflammatory state, amplifying systemic inflammation through IL-6 upregulation and thereby promoting the development of conditions such as insulin resistance and atherosclerosis [141]. The leptin-IL-6 relationship is among the better-supported adipokine-cytokine associations in obesity, with consistent positive correlations documented in multiple human cross-sectional studies. However, causality remains difficult to establish in human cohorts because both leptin and IL-6 rise in parallel with adiposity and systemic inflammation, precluding a clear determination of whether hyperleptinemia drives IL-6 elevation or vice versa. Interventional studies measuring IL-6 before and after pharmacological leptin receptor modulation in humans are needed to confirm the directionality implied by preclinical mechanistic data. Effects of Leptin on IL-6 are illustrated in Fig. 2B.

#### Effect of Leptin on IL-8

Within pro-inflammatory and pro-malignant contexts, leptin directly upregulates the potent chemokine IL-8 (CXCL8). This effect, characterized by concentration- and time-dependent increases in IL-8 transcript and protein, has been validated in multiple cancer and stromal cell types [142]. The mechanism involves Ob-R receptor signaling through PI3K/Akt and MAPK, culminating in NF-κB activation and IL-8 transcription [143]. This cascade functionally contributes to oncogenesis by enhancing EMT and invasive capacity, providing a molecular bridge from hyperleptinemia to cancer progression [143]. The mechanistic evidence linking leptin to IL-8 induction currently rests primarily on in vitro experiments in cancer cell lines and synovial fibroblast models; in vivo confirmation of this pathway in obesity-associated tumor models, and human data demonstrating that hyperleptinemia elevates tumor-associated IL-8 levels, are lacking. This pathway should therefore be regarded as a biologically plausible mechanistic hypothesis supported by cell-based evidence, rather than an established link in human obesity-associated oncogenesis. Effects of Leptin on IL-8 are illustrated in Fig. 2B.

#### Effect of Leptin on MCP-1

The activation of leptin-driven signaling cascades (JAK-STAT3, MAPK/ERK, PI3K-Akt) results in a pro-inflammatory TME [144]. This chemokine milieu drives the infiltration of immune cells that contribute to tumorigenesis by promoting angiogenesis and sustaining proliferation [145, 146].

Leptin signaling promotes key hallmarks of cancer progression often in conjunction with elevated MCP-1 expression, suggesting a functional interplay between these pathways [147]. The reprogramming of TAMs toward an M1-like phenotype by leptin leads to elevated MCP-1 levels and a more immunosuppressive TME. On the other hand, it was found that MCP-1 expression is positively associated with CD68 or CD163 expression, marker of M2 macrophage, in DLBCL [112]. Transcript levels of both M1-like (CD11c, iNOS) and M2-like (CD206) macrophage markers were increased in the mammary glands of mice fed a high-fat diet [148]. These changes collectively promote tumor aggressiveness and can compromise the efficacy of immunotherapeutic interventions [149]. An unresolved controversy regarding leptin’s effect on MCP-1 concerns the net polarization outcome of the macrophages recruited by this axis. Some preclinical studies describe leptin-driven MCP-1 upregulation as promoting M1-like macrophage recruitment with pro-inflammatory cytokine secretion, while others describe the recruited TAMs as adopting an M2-like, immunosuppressive phenotype, consistent with the broader TAM literature [150–152]. This apparent contradiction likely reflects cancer-type and microenvironment-specific differences in macrophage programming, and direct single-cell resolution data from human obese tumor tissue are needed to characterize the dominant macrophage phenotype recruited by the leptin-MCP-1 axis in vivo. Effects of Leptin on MCP-1 are illustrated in Fig. 2B.

#### Effect of Leptin on IL-1β

In breast and ovarian cancer cells, leptin directly stimulates the expression of IL-1β and related IL-1 system components (e.g., IL-1α, IL-1Ra, IL-1RI) at the transcriptional and translational levels. This induction is mediated through multiple signaling pathways, such as JAK2/STAT3, MAPK/ERK, and PI3K/AKT. Consequently, leptin-driven IL-1β production constitutes a critical element of a pro-inflammatory and pro-angiogenic program that fuels tumor progression [153, 154].

Leptin-driven upregulation of IL-1β subsequently enhances the expression of VEGF and its receptor VEGFR2, thereby promoting angiogenesis, which is critical for tumor growth and expansion [154]. The functional importance of this axis is demonstrated by the fact that inhibition of IL-1 signaling significantly attenuates leptin-induced VEGF/VEGFR2 upregulation [155, 156]. In breast and hepatic cancer cells, leptin stimulates IL-1β maturation and inflammasome activation, processes that are mechanistically linked to enhanced tumor growth [157, 158]. The pro-tumorigenic effects of this pathway can be attenuated by the adipokine adiponectin or through the induction of the cytoprotective HO-1 pathway [159]. Effects of Leptin on IL-1β are illustrated in Fig. 2B.

### Effect of Adiponectin on SASPs

**Fig. 3:**
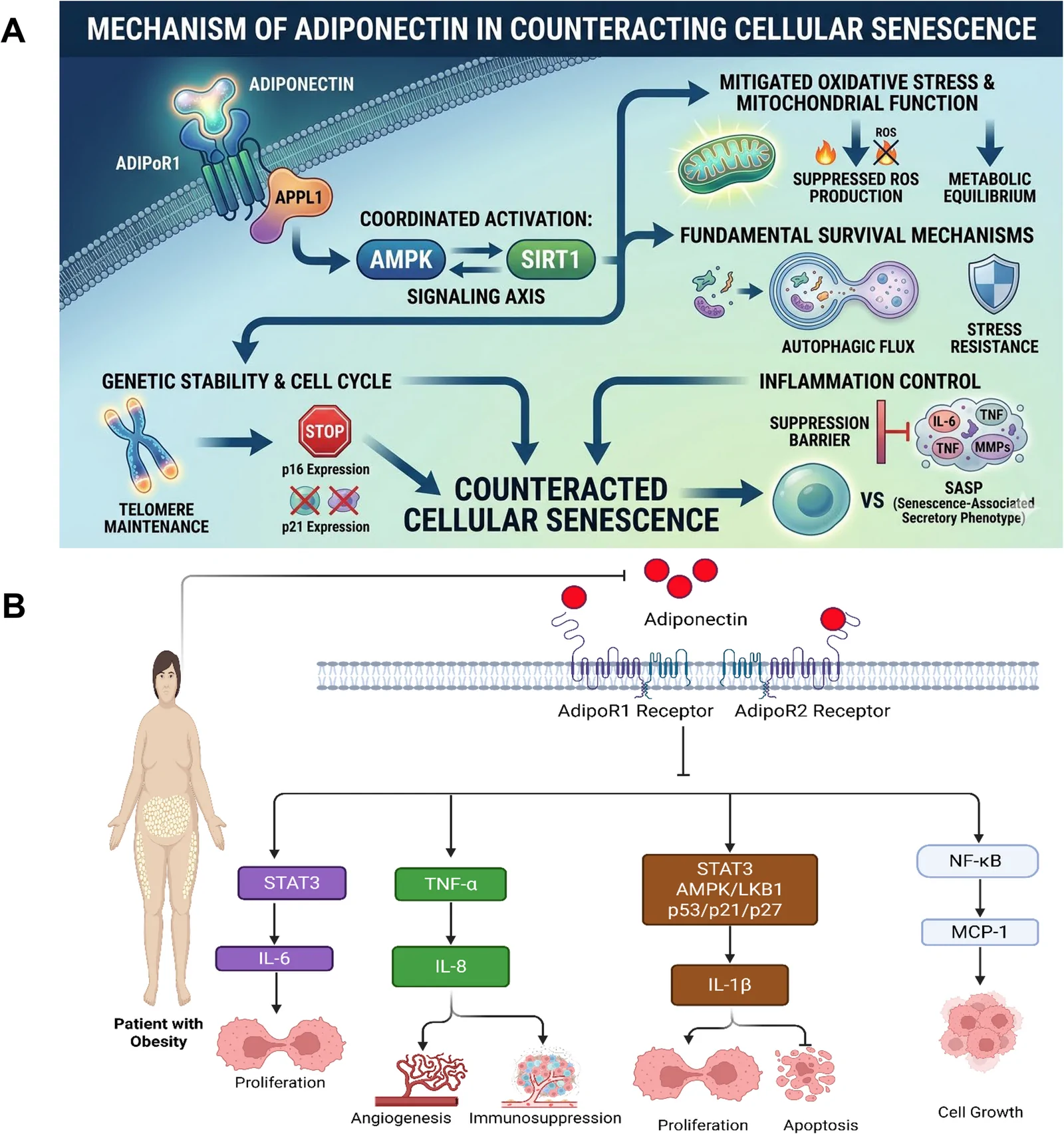
(**A**) Adiponectin signaling counteracts cellular senescence through coordinated AMPK–SIRT1 activation and metabolic protection. Schematic representation of the molecular mechanisms by which adiponectin mitigates cellular senescence. Binding of adiponectin to its receptor AdipoR1 recruits the adaptor protein APPL1, leading to coordinated activation of the AMPK–SIRT1 signaling axis. This pathway promotes mitochondrial function and reduces oxidative stress, resulting in suppressed ROS production and maintenance of metabolic homeostasis. Concurrently, adiponectin enhances fundamental survival mechanisms, including increased autophagic flux and improved cellular stress resistance. At the genomic level, adiponectin preserves telomere integrity and regulates cell cycle progression through suppression of senescence-associated cyclin-dependent kinase inhibitors, including p16 and p21. Additionally, activation of this pathway attenuates inflammatory signaling by limiting the senescence-associated secretory phenotype (SASP), including pro-inflammatory cytokines and matrix remodeling factors. (**B**) Schematic representation of adiponectin signaling and its dysregulation in obesity, highlighting downstream effects on inflammation, tumor progression, and cellular responses. In obese individuals, reduced adiponectin levels limit activation of AdipoR1 and AdipoR2 receptors, leading to stimulation of STAT3 and TNF‑α pathways, resulting in increased IL‑6 and IL‑8 production, which promote tumor cell proliferation, angiogenesis, and immunosuppression. Additional signaling via STAT3/AMPK/LKB1 and p53/p21/p27 pathways regulates IL‑1β expression, influencing proliferation and apoptosis. Concurrent activation of NF‑κB signaling increases MCP‑1 production, further supporting tumor cell growth. Collectively, these pathways illustrate how impaired adiponectin signaling in obesity contributes to a pro-tumorigenic microenvironment. AMPK: adenosine monophosphate-activated protein kinase, APPL1: Adaptor protein, phosphotyrosine interacting with PH domain and leucine zipper 1, ERK: Extracellular signal-Regulated Kinase, IL-1β: Interleukin-1 beta, IL-6: Interleukin 6, IL-8: Interleukin 8, LKB1: Liver kinase B1, MCP-1: Monocyte Chemoattractant Protein-1, NF-κB: Nuclear Factor kappa B, SASP: Senescence-Associated Secretory Phenotype, SIRT1: Sirtuin 1, STAT: Signal Transducer and Activator of Transcription, TNF-α: Tumor Necrosis Factor-alpha

Adiponectin counteracts cellular senescence by mitigating oxidative stress, primarily through the suppression of ROS production [160, 161]. The coordinated activation of AMPK, SIRT1, and AdipoR1/APPL1 pathways by adiponectin enhances fundamental survival mechanisms, such as autophagic flux, metabolic equilibrium, and stress resistance [162, 163]. Furthermore, adiponectin counteracts senescence through a multi-faceted approach: it suppresses the expression of key markers (p16, p21) and the SASP while promoting telomere maintenance and enhancing mitochondrial function [163–165]. Mechanism of adiponectin signaling counteracts cellular senescence is illustrated in Fig. 3A. The anti-senescent and anti-SASP properties of adiponectin described in this section are well documented at the mechanistic level, and the inverse association between circulating adiponectin and cancer risk has been consistently observed across epidemiological studies of breast [166, 167], colorectal [168, 169], and endometrial cancers [170] in human cohorts. However, a critical translational challenge is that adiponectin’s multimeric complexity, with distinct biological activities associated with trimeric, hexameric, and high-molecular-weight forms, means that total circulating adiponectin measurements may not accurately reflect the biologically active fraction relevant to cancer risk. This complicates both the interpretation of epidemiological associations and the development of therapeutic adiponectin-based strategies.

Collectively, these findings suggest adiponectin as a multifunctional brake on the senescence program: by simultaneously dampening oxidative stress, sustaining autophagic flux, suppressing SASP marker expression, and preserving telomere integrity, adiponectin counteracts each of the principal mechanisms through which obesity drives adipose tissue senescence. Its deficiency in obesity therefore creates a permissive environment in which SASP amplification proceeds unchecked, directly linking hypoadiponectinemia to the pro-tumorigenic remodeling of the tissue microenvironment. Effects of adiponectin on SASPs are illustrated in Fig. 3B.

#### Effect of Adiponectin on IL-6

Adiponectin functions as an anti-inflammatory and anti-cancer agent, primarily by suppressing the production and signaling of IL-6 and other pro-inflammatory cytokines in immune cells like macrophages. This action attenuates the tumor-promoting inflammatory milieu, thereby impeding cancer progression [171]. Adiponectin and IL-6 exert counteracting influences on tumor behavior; high adiponectin levels correlate with suppressed proliferation, metastasis, and better survival, in contrast to the pro-tumorigenic effects of IL-6 in colorectal cancer [172]. This improved prognosis associated with adiponectin is likely mediated by its inhibition of IL-6 signaling in nasopharyngeal carcinoma [173]. By reducing STAT-3 phosphorylation and activation, adiponectin administration on MC-38 cells inhibited IL-6-induced cell proliferation [172]. Taken together, these findings establish adiponectin as a suggested antagonist of IL-6-driven oncogenesis: it suppresses IL-6 production by macrophages [171], disrupts IL-6/STAT3 signaling in tumor cells [174], and thereby reverses the proliferative and survival advantages that IL-6 confers on malignant cells. In the context of obesity where adiponectin is chronically depressed and IL-6 chronically elevated, this antagonism is lost, effectively removing a key endogenous brake on tumor-promoting inflammation and positioning hypoadiponectinemia as a direct mechanistic contributor to obesity-associated cancer progression. Effects of adiponectin on IL-6 are illustrated in Fig. 3B.

#### Effect of Adiponectin on IL-8

Adiponectin exerts an anti-inflammatory effect on vascular endothelium by suppressing TNF-α-induced IL-8 production [175]. The mechanism involves dual action: inhibition of the NF-κB pathway and cAMP/PKA-dependent activation of Akt, leading to reduced IL-8 expression and secretion [176]. In the context of obesity-associated cancer, this adiponectin-mediated restraint on IL-8 is mechanistically critical: as established in Sect. Effect of IL-8 on cancer progression, IL-8 drives tumor angiogenesis [177, 178], recruits immunosuppressive myeloid populations [179, 180], and promotes metastatic dissemination [177, 178]. The obesity-driven decline in adiponectin therefore removes a dual vascular and immunological restraint, permitting IL-8 to fuel tumor neovascularization and therapeutic resistance within an inflammatory microenvironment that is already permissive to cancer progression. Effects of adiponectin on IL-8 are illustrated in Fig. 3B.

#### Effect of Adiponectin on MCP-1

A key anti-cancer mechanism of adiponectin involves the suppression of pro-inflammatory mediators like MCP-1, leading to the inhibition of cancer cell growth. This is exemplified in lung epithelial cells, where adiponectin attenuates MCP-1 production, an effect potentially mediated through the inhibition of the master inflammatory regulator NF-κB [181, 182]. This suppression of MCP-1 is of direct relevance within the framework of this review: MCP-1 is the primary recruiter of circulating monocytes into the tumor-adjacent adipose stroma, where they differentiate into pro-tumorigenic TAMs as described in Sect. Introduction. By restraining MCP-1 through NF-κB inhibition [183, 184], adiponectin normally limits this macrophage recruitment axis. Hypoadiponectinemia in obesity therefore disinhibits NF-κB [185], permitting unchecked MCP-1-driven monocyte infiltration and perpetuating the immunosuppressive CLSs environment characteristic of patients with obesity adipose tissue — a microenvironment that directly feeds the SASP-driven pro-tumorigenic niche. Effects of adiponectin on MCP-1 are illustrated in Fig. 3B.

#### Effect of Adiponectin on IL-1β

Adiponectin counteracts IL-1β-mediated oncogenesis by inhibiting cancer cell proliferation and inflammation [172, 173, 182]. In colon cancer, this protective effect—synergized by metformin—operates through STAT3 and AMPK/LKB1 pathways to restore cell cycle control by upregulating p53/p21/p27 and suppressing cyclins [186]. Raut and Park [159] showed that Globular adiponectin inhibits tumor growth by suppressing inflammasome activation (IL-1β/caspase-1) in breast and liver cancer cells, leading to reduced proliferation and increased apoptosis via HO-1 and estrogen receptor-α pathways. Nigro, Scudiero [182] Showed that via the AdipoR1 receptor, adiponectin counteracts IL-1β-induced damage in lung epithelial cells by promoting viability, suppressing apoptosis and NF-κB signaling, and selectively upregulating anti-inflammatory IL-10.

Collectively, these data demonstrate that adiponectin suppresses IL-1β-mediated oncogenesis through potential complementary upstream and downstream mechanisms: blockade of inflammasome activation, inhibition of NF-κB-driven inflammatory transcription, and restoration of tumor-suppressive cell cycle checkpoints via p53/p21/p27 upregulation. In obesity, where adiponectin is deficient and the NLRP3 inflammasome may be constitutively primed by metabolic danger signals, this protective circuit is disrupted — allowing IL-1β contribution to propagate the SASP, drive EMT, stimulate angiogenesis, and suppress anti-tumor immunity without restraint. Adiponectin thus functions as a potential physiological senomorphics agent, and its loss in obesity may represent a key vulnerability through which the SASP-driven pro-tumorigenic program is licensed to proceed.

Adiponectin’s modulation of all four SASP cytokines in obesity suggests a compelling mechanistic case for hypoadiponectinemia as a critical permissive factor in obesity-associated oncogenesis. The evidence is strongest for the adiponectin-IL-6 and adiponectin-IL-1β axes, where both mechanistic pathway data and human epidemiological correlations converge. The adiponectin-IL-8 and adiponectin-MCP-1 evidence bases are more limited and predominantly mechanistic. A broader unresolved controversy is whether the cancer-preventive effects attributed to adiponectin in epidemiological studies are causally driven by adiponectin itself or confounded by correlated variables such as insulin sensitivity, physical activity, and overall metabolic health — all of which track with adiponectin levels and independently affect cancer risk. Effects of adiponectin on IL-1β are illustrated in Fig. 3B.

### Effect of Insulin on SASPs

The case for insulin and hyperglycemia as drivers of SASP amplification is mechanistically strong, with the NLRP3/IL-1β axis providing a potential well-characterized molecular link supported by both cell-based and in vivo evidence. However, a key unresolved question is how much of the cancer risk attributable to insulin resistance is mediated by SASP amplification specifically, versus direct mitogenic IGF-1R/AKT/mTOR signaling on tumor cells, which may operate independently of SASP and has a substantial literature of its own. Disentangling these two routes of insulin-associated cancer promotion is clinically important because they imply different therapeutic strategies: SASP suppression versus IGF-1R blockade versus metabolic normalization.

**Fig. 4:**
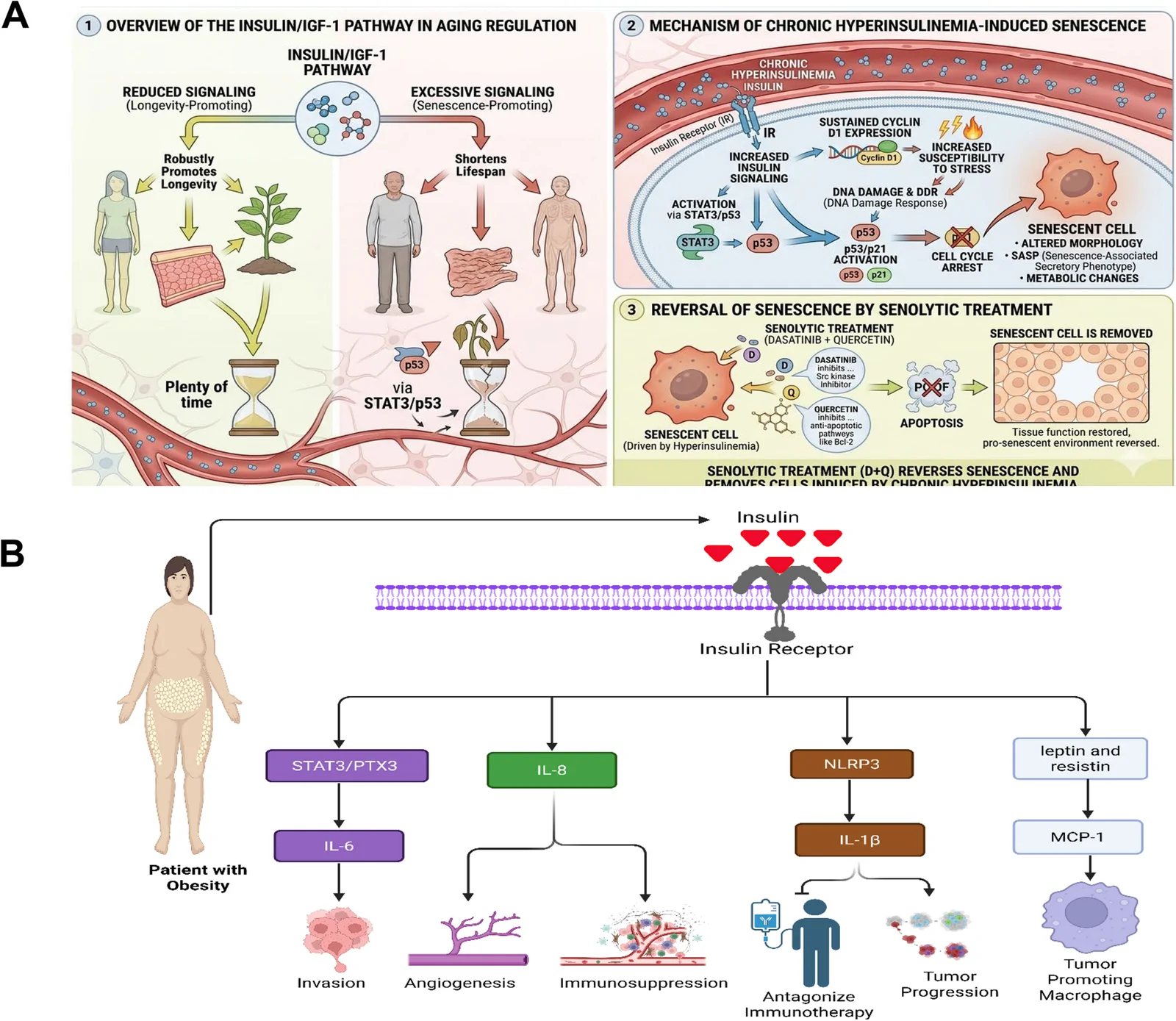
(**A**) Insulin/IGF‑1 signaling in aging and the role of chronic hyperinsulinemia in driving cellular senescence, with reversal by senolytic therapy. (1) Overview of the insulin/IGF‑1 pathway in aging regulation. Balanced or reduced insulin/IGF‑1 signaling promotes longevity, partly through stress resistance pathways including STAT3 and p53. In contrast, excessive signaling accelerates aging, shortens lifespan, and promotes tissue dysfunction. (2) Mechanism of chronic hyperinsulinemia‑induced senescence. Sustained insulin exposure enhances insulin receptor (IR) signaling, leading to increased activation of STAT3 and p53/p21 pathways. This signaling cascade induces DNA damage responses, cell cycle arrest, and increased susceptibility to cellular stress. These events culminate in the development of a senescence characterized by altered morphology, metabolic dysfunction, and acquisition of a senescence‑associated secretory phenotype (SASP). (3) Reversal of senescence by senolytic therapy. Treatment with senolytic agents (Dasatinib and Quercetin, D + Q) selectively induces apoptosis in senescent cells, thereby reducing senescent cell burden. Clearance of senescent cells restores tissue function and reverses the pro‑senescent environment induced by chronic hyperinsulinemia. (**B**) Schematic overview of insulin signaling in obesity and its downstream effects on inflammation, tumor progression, and immune modulation. In obese individuals, elevated insulin levels activate the insulin receptor, triggering multiple signaling pathways including STAT3/PTX3, IL‑8, and NLRP3 axes. STAT3/PTX3 signaling induces IL‑6 production, promoting tumor invasion, while IL‑8 enhances angiogenesis and immunosuppression. Activation of the NLRP3 inflammasome increases IL‑1β levels, contributing to tumor progression and reduced responsiveness to immunotherapy. Additionally, insulin-associated adipokines such as leptin and resistin upregulate MCP‑1, facilitating the recruitment of tumor-promoting macrophages. Collectively, these pathways highlight the role of hyperinsulinemia in shaping a pro-tumorigenic microenvironment. IGF-1: Insulin-like Growth Factor 1, IL-1β: Interleukin-1 beta, IL-6: Interleukin 6, IL-8: Interleukin 8, MCP-1: Monocyte Chemoattractant Protein-1, NLRP3: NOD-, LRR- and pyrin domain-containing protein 3, PTX3: Pentraxin 3, STAT: Signal Transducer and Activator of Transcription

Baboota, Spinelli [187] showed that chronic hyperinsulinemia drives hepatocyte senescence through p53/p21 activation and increased susceptibility to stress, mediated in part by sustained Cyclin D1 expression. This pro-senescent effect is reversible by senolytic treatment with dasatinib and quercetin. The translational potential of senolytic strategies has begun to be evaluated in early-phase human studies. A first-in-human pilot trial of the dasatinib plus quercetin (D + Q) combination in patients with idiopathic pulmonary fibrosis, a senescence-driven fibrotic disease, demonstrated selective reduction of senescent cell burden in adipose tissue and skin biopsies, alongside improvements in physical function, providing proof-of-concept for the clinical feasibility of pharmacological senescent cell clearance in humans [188]. In the oncology context, D + Q and the senolytic navitoclax (ABT-263) are currently under investigation in early-phase clinical trials for their capacity to improve responses to chemotherapy and immunotherapy in solid tumors. Studies such as NCT01557777 investigates the effect of navitoclax on subjects with chronic lymphocytic leukemia (CLL) while NCT03366103 study effect of navitoclax and vistusertib in treating patients with relapsed small cell lung cancer and other solid tumors, however, critical translational challenges remain: (i) the absence of validated, tumor-specific SASP biomarkers for patient selection and pharmacodynamic monitoring; (ii) the risk of on-target toxicity from broad senescent cell clearance, which could impair wound healing and tissue homeostasis; and (iii) the difficulty of distinguishing obesity-driven senescence burden from age-related senescence in clinical populations, which complicates trial design. These challenges highlight that while senolytics represent a scientifically compelling strategy for interrupting the obesity-SASP-cancer axis, their clinical application in oncology remains at an early stage and requires both biomarker development and carefully designed combination trial strategies. Insulin/IGF‑1 signaling in aging and the role of chronic hyperinsulinemia in driving cellular senescence is illustrated in Fig. 4A.

#### Effect of Insulin on IL-6

A pathogenic cycle links insulin resistance, IL-6, and cancer, particularly in obesity. AT-derived IL-6 impairs insulin signaling, fostering hyperinsulinemia, which in turn can promote tumor growth [189, 190]. Concurrently, IL-6 directly shapes a pro-tumorigenic microenvironment. This vicious cycle is evidenced by the strong association between elevated IL-6 levels and an increased risk of cancer [191]. Xie, Ruan [192] Showed that the enhanced invasiveness was attributable to elevated IL-6, which was secreted by mature adipocytes in culture and present at higher concentrations in the serum of obese mice. The functional role of IL-6 was confirmed by treating cells with the recombinant cytokine, which directly stimulated triple-negative breast cancer (TNBC) cell migration and invasion and activated the STAT3/PTX3 signaling axis [193]. These findings reveal a self-reinforcing pathological circuit uniquely amplified in obesity: adipose-derived IL-6, driven in part by the hyperinsulinemia state, may worsen insulin resistance while simultaneously activating oncogenic STAT3 signaling in neighboring cancer cells. This bidirectional crosstalk means that metabolic and oncogenic dysfunction are not merely parallel consequences of obesity but mutually reinforcing processes, and that IL-6 functions as a potential molecular bridge between systemic metabolic disorder and the local pro-tumorigenic microenvironment — a mechanistic insight that directly implicates the hyperinsulinaemia-IL-6-SASP axis as a therapeutic target in obesity-associated cancers. Effects of Insulin on IL-6 are illustrated in Fig. 4B.

#### Effect of Insulin on IL-8

Insulin acts synergistically with fatty acids (e.g., palmitate) to upregulate the pro-tumorigenic chemokine IL-8 in macrophages, thereby promoting processes critical for cancer progression, like angiogenesis and metastasis [194, 195]. In vitro, insulin synergizes with palmitate to markedly enhance IL-8 secretion, beyond its modest effect on mRNA alone, mimicking the amplified inflammatory response seen in metabolic dysfunction [196]. This synergy between hyperinsulinemia and dyslipidemia in driving macrophage IL-8 production is particularly consequential in obesity, where both conditions chronically co-exist. The resulting IL-8 surge promotes tumor angiogenesis [197, 198], recruits immunosuppressive myeloid populations to the TME [179, 180], and generates a chemotactic environment favoring metastatic dissemination — effects that neither metabolic perturbation would produce in isolation. This observation highlights that the suggested pro-tumorigenic impact of obesity on the SASP is not simply additive across individual metabolic derangements, but is qualitatively amplified by their convergence, reinforcing the importance of simultaneously addressing multiple metabolic risk factors in obesity-associated cancer prevention and management. Effects of Insulin on IL-8 is illustrated in Fig. 4B.

#### Effect of Insulin on MCP-1

Sartipy and Loskutoff [199] results suggest that the effect of insulin on MCP-1 is determined by metabolic status: it increases MCP-1 in AT of insulin-resistant individuals, potentially worsening inflammation, but decreases serum MCP-1 in insulin-sensitive individuals. Elevated MCP-1 itself may promote adipocyte dysfunction and contribute to diabetic pathogenesis [200, 201]. Through elevated levels of leptin and resistin, MCP-1 in type 2 diabetes exacerbates insulin resistance and decreased glucose tolerance [202]. Critically, these findings reveal that MCP-1 is not merely a downstream effector of insulin resistance but an active perpetuator of it: once elevated by obesity, hyperinsulinemia state, MCP-1 may drive monocyte infiltration into adipose tissue, further worsening local inflammation and insulin signaling. This feed-forward loop in which metabolic dysfunction elevates MCP-1 and MCP-1 deepens metabolic dysfunction, may simultaneously amplifies the SASP, sustains CLSs formation, and continuously recruits tumor-promoting macrophages into the adipose and peritumoral microenvironment, cementing MCP-1 as a potential important mechanistic link between insulin resistance and obesity-driven oncogenesis [199, 202]. Effects of Insulin on MCP-1 is illustrated in Fig. 4B.

#### Effect of Insulin on IL-1β

The metabolic dysfunction of high insulin and glucose promotes cancer by upregulating IL-1β. In cholangiocarcinoma (bile duct cancer), Khawkhiaw, Chomphoo [203] showed that in tumor cells, hyperglycemia promotes IL-1β expression, which in turn drives proliferation and aggression. The reversibility of this effect by IL-1β blockade confirms a causative role for this cytokine in metabolically driven cancer progression [204].

IL-1β orchestrates key pro-tumorigenic processes including chronic inflammation, angiogenesis, cancer cell proliferation and dissemination, and immunosuppression [205–207]. In preclinical studies, IL-1β blockade with receptor antagonists not only suppresses tumor progression but also synergizes with immunotherapies, highlighting its potential as a combination strategy [208]. Metabolic disturbances characterized by high insulin and glucose promote cancer by upregulating IL-1β. This mechanistic link positions IL-1β signaling as a compelling therapeutic target for metabolically associated cancers [209].

The most clinically significant human evidence for IL-1β blockade in the cancer-metabolism interface comes from the CANTOS (Canakinumab Anti-inflammatory Thrombosis Outcomes Study) trial, a landmark randomized controlled trial of the anti-IL-1β monoclonal antibody canakinumab in 10,061 patients with prior myocardial infarction and elevated C-reactive protein, a population enriched for metabolic inflammation [210]. Strikingly, canakinumab significantly reduced incident lung cancer and cancer-related mortality [211]. This finding provided the first large-scale human evidence that systemic IL-1β suppression can reduce cancer incidence in a metabolically dysregulated population, directly validating the mechanistic framework presented in this review. Ongoing trials including Canakinumab (NCT03626545) and combination studies pairing IL-1β blockade with Docetaxel in NSCLC and other solid tumors will help define the therapeutic window and optimal combinatorial context for IL-1β-targeted strategies in obesity-associated cancers.

Beyond pharmacological SASP targeting, metabolic interventions that directly address the underlying drivers of hyperinsulinemia and adipose tissue inflammation represent a clinically actionable and evidence-backed strategy for disrupting the obesity-SASP-cancer axis. Metformin, the most widely prescribed insulin-sensitizing agent, has shown consistent associations with reduced cancer incidence and improved oncological outcomes in retrospective and observational studies of patients with type 2 diabetes, with particular evidence in colorectal, breast, and endometrial cancers [212, 213]. The proposed mechanisms include AMPK activation leading to mTOR suppression and reduced SASP secretion, direct anti-proliferative effects via cell cycle arrest, and reduction of hyperinsulinemia and IGF-1 signaling.

Integrating these observations, hyperinsulinemia and hyperglycemia act as metabolic primers of the NLRP3 inflammasome, establishing a potential state of constitutive IL-1β activation in patients with obesity adipose and hepatic tissue. This chronically activated IL-1β axis propagates the SASP by sustaining NF-κB-driven cytokine transcription, fuels cancer progression through EMT, angiogenesis, and immune evasion, and simultaneously deepens insulin resistance through a feedback loop that further elevates glucose and insulin levels. In the context of obesity-associated cancer, metabolic dysfunction and SASP amplification are therefore not sequentially ordered events but simultaneous, mutually reinforcing processes — an integrated pathological circuit in which IL-1β occupies a central node with both mechanistic and therapeutic significance. Effects of Insulin on IL-1β is illustrated in Fig. 4B.

### Effect of Resistin on SASPs

**Fig. 5:**
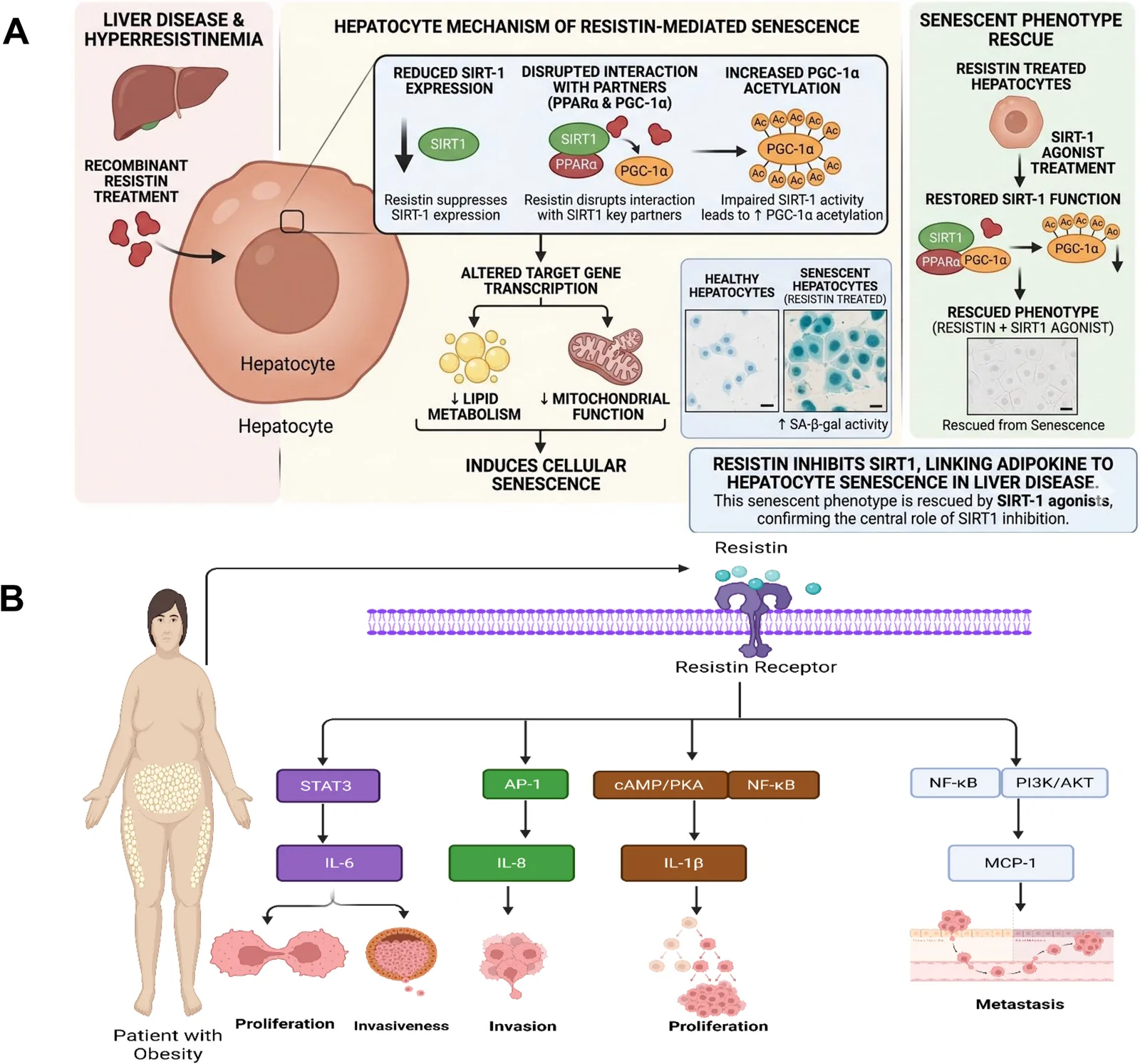
(**A**) Resistin promotes hepatocyte senescence through SIRT1 inhibition and disrupted metabolic signaling. Schematic illustration of the mechanism by which hyperresistinemia in liver disease induces hepatocyte senescence. Recombinant resistin treatment reduces SIRT1 expression and disrupts its interaction with key metabolic regulators, including PPARα and PGC‑1α. Impaired SIRT1 activity leads to increased acetylation of PGC‑1α, resulting in altered transcription of target genes that regulate lipid metabolism and mitochondrial function. These metabolic alterations promote cellular senescence, as evidenced by increased senescence-associated β‑galactosidase (SA‑β‑gal) activity in resistin-treated hepatocytes compared to healthy controls. Pharmacologic activation of SIRT1 restores its function, re-establishes interactions with metabolic partners, and reverses the senescent phenotype. Collectively, these findings identify SIRT1 inhibition as a central mechanism linking resistin signaling to hepatocyte senescence in liver disease and suggest that SIRT1 activation may represent a therapeutic strategy to rescue resistin-induced dysfunction. (**B**) Schematic illustration of resistin-mediated signaling pathways in obesity and their effects on tumor progression. In obese individuals, resistin binding to its receptor activates multiple downstream pathways, including STAT3, AP‑1, cAMP/PKA, NF‑κB, and PI3K/AKT. These signaling cascades induce the production of pro-inflammatory cytokines such as IL‑6, IL‑8, IL‑1β, and MCP‑1. IL‑6 promotes tumor cell proliferation and invasiveness, while IL‑8 enhances invasion. IL‑1β further supports tumor cell proliferation, and MCP‑1 contributes to metastatic dissemination via NF‑κB and PI3K/AKT signaling. Collectively, these pathways highlight the role of resistin in promoting inflammation-driven tumor growth and metastasis in obesity. AKT: Protein Kinase B, AP-1: Activator Protein-1, cAMP: cyclic adenosine monophosphate, IL-1β: Interleukin-1 beta, IL-6: Interleukin 6, IL-8: Interleukin 8, MCP-1: Monocyte Chemoattractant Protein-1, NF-κB: Nuclear Factor kappa B, PGC-1α: Peroxisome proliferator-activated receptor gamma coactivator 1-alpha, PI3K: Phosphoinositide 3-kinase, PKA: Protein Kinase A, PPAR-α: Peroxisome Proliferator-Activated Receptor Alpha, SIRT1: Sirtuin 1, STAT: Signal Transducer and Activator of Transcription

The inhibition of SIRT1 by resistin in hepatocytes identifies a potential mechanistic link between this adipokine and the onset of cellular senescence in liver disease [214] .Yu, Zheng [214] show that Treatment with recombinant resistin impairs SIRT-1 function in hepatocytes by reducing its expression, disrupting its interaction with key partners like PPARα and PGC-1α, and increasing PGC-1α acetylation. This suppression of SIRT-1 activity alters the transcription of its target genes and, critically, induces cellular senescence, as evidenced by increased SA-β-gal activity. The senescent phenotype is rescued by SIRT-1 agonists, confirming the central role of SIRT1 inhibition in resistin-mediated senescence. An important species-specific caveat must be acknowledged when interpreting the resistin literature: unlike leptin and adiponectin, resistin is produced predominantly by adipocytes in rodents, but in humans it is secreted primarily by monocytes and macrophages [215, 216]. This fundamental biological difference means that mechanistic data generated in murine models may not directly translate to human obesity-associated physiology, where resistin’s cellular sources and regulation are fundamentally different. Resistin induction of hepatocyte senescence is illustrated in Fig. 5A.

In the context of obesity-associated cancer, this resistin–SIRT1–senescence axis carries a critical implication: SIRT1 normally deacetylates and inactivates both NF-κB [217] and p53 [218], thereby restraining SASP factor transcription and maintaining cellular homeostasis. Resistin-mediated SIRT1 suppression therefore may simultaneously unleashes NF-κB-driven SASP amplification and dysregulates p53-dependent tumor suppression — providing a plausible molecular explanation for the epidemiological link between elevated resistin, hepatic steatosis, and HCC risk observed in individuals with obesity. All effects of resistin on SASPs are illustrated in Fig. 5.

#### Effect of Resistin on IL-6

Clinically, Kallio, Hämäläinen [219] showed that elevated resistin and IL-6 levels correlate with advanced disease and poor survival in cancers such as renal cell and breast carcinoma. Mechanistically, resistin directly upregulates IL-6 expression in cancer cells, with a particularly potent effect in models derived from African American patients, mirroring higher serum levels observed in this population. This resistin-IL-6 axis drives tumor aggressiveness by activating STAT3, as confirmed by the abolition of STAT3 phosphorylation upon IL-6 blockade, establishing a clear signaling hierarchy [220]. These findings are significant within the broader framework of this review because it position resistin as a potential upstream adipokine regulator of the IL-6/STAT3 axis — already established as a principal mediator of tumor proliferation, invasion, and immune evasion in Sect. Effect of IL-6 on cancer progression. In obesity, where circulating resistin is chronically elevated, this resistin–IL-6–STAT3 hierarchy provides a suggested additional and independent hormonal input that sustains pro-tumorigenic SASP signaling, operating in parallel with hyperleptinemia and hyperinsulinemia. This convergence of multiple obesity-associated adipokines on a shared oncogenic pathway underscores the difficulty of interrupting SASP-driven cancer promotion at the level of individual cytokines and suggests that upstream adipokine normalization may offer greater therapeutic leverage. Effects of Resistin on IL-6 is illustrated in Fig. 5B.

#### Effect of Resistin on IL-8

A key mechanism of resistin action is the direct activation of AP-1, which drives the transcription of pro-inflammatory, pro-adhesive, and vascular remodeling genes [221]. Resistin promotes a robust pro-inflammatory response by activating the AP-1 transcription factor, leading to the upregulation of key cytokines such as TNF-α, IL-1β, IL-6, IL-8, and IL-12 [222]. In the context of this review, resistin’s activation of AP-1 is particularly noteworthy because it positions resistin as a potential broad-spectrum transcriptional inducer of SASP components rather than a cytokine-specific regulator: a single adipokine elevated in obesity simultaneously upregulates IL-6, IL-8, IL-1β, and TNF-α through one shared transcription factor [222]. This mechanistic convergence on AP-1 means that in the patients with obesity adipose microenvironment, resistin may function as a master switch amplifying multiple arms of the SASP in parallel, with the combined cytokine output driving immune evasion, tumor angiogenesis, and cancer cell invasion more effectively than any individual SASP component would in isolation. Effects of Resistin on IL-8 is illustrated in Fig. 5B.

#### Effect of Resistin on MCP-1

The inflammatory adipokine resistin contributes to tumor progression by enhancing the pro-tumorigenic microenvironment, in part through the upregulation of chemokines such as MCP-1 [223]. Clinically, elevated resistin levels are correlated with advanced disease characteristics, including larger tumor size, higher grade, and increased metastasis in breast cancer [224, 225]. Although direct evidence for resistin-induced MCP-1 in cancer is still emerging, resistin’s established role as a pro-inflammatory mediator and its capacity to activate key signaling pathways such as NF-κB and PI3K/AKT, both known regulators of MCP-1, provide a strong mechanistic rationale for this connection [121]. While direct causal evidence for resistin-driven MCP-1 induction in obesity-associated tumors is still emerging, the convergence of NF-κB and PI3K/AKT signaling as potential shared downstream effectors of both resistin and MCP-1 regulation suggests that hyperresistinemia in obesity contributes to MCP-1-mediated macrophage recruitment into the tumor microenvironment. If confirmed, this would identify resistin as an adipokine that amplifies the SASP not only through direct cytokine induction but also by reinforcing the macrophage infiltration axis that sustains chronic adipose tissue inflammation and TAM polarization creating a self-perpetuating cycle that links adipokine dysregulation in obesity to immunological tumor promotion. Effects of Resistin on MCP-1 is illustrated in Fig. 5B.

#### Effect of Resistin on IL-1β

The pro-inflammatory actions of resistin, such as the induction of IL-1β, IL-6, and TNF-α, are mediated through the CAP1 receptor [226]. Ligand binding triggers a cAMP/PKA signaling cascade that activates NF-κB and enhances the transcription of target cytokines [227]. Resistin promotes a pro-tumorigenic feedback loop by activating NF-κB in macrophages to induce IL-1β production. This, in turn, drives the recruitment of inflammatory cells and further cytokine secretion, creating a self-reinforcing cycle of chronic inflammation that fuels cancer progression [228, 229]. This resistin–CAP1–NF-κB–IL-1β circuit is particularly meaningful in the context of obesity-associated cancer because it may link the adipokine excess of obesity directly to the most potent SASP inflammasome axis reviewed in this article. Elevated resistin activates NF-κB [229] to induce IL-1β [230], which in turn activates NF-κB [231] further and primes NLRP3 inflammasome assembly [232, 233], creating a self-amplifying inflammatory loop that is independent of direct tumor cell input and can therefore be established and maintained by the patients with obesity adipose microenvironment alone. This provides a suggested mechanistic basis for the contribution of obesity to cancer initiation not merely progression and positions the resistin–IL-1β axis as a pre-tumorigenic therapeutic target in individuals with obesity who have not yet developed malignancy.

In summary, the mechanistic evidence for resistin as a SASP amplifier is consistent across its four cytokine targets but rests predominantly on in vitro cell-based studies and animal model data. Human clinical evidence linking circulating resistin levels to SASP marker concentrations or cancer outcomes is limited and, in the case of cancer risk, has produced inconsistent findings across studies, with some cohort analyses reporting a positive association between resistin and cancer risk while others have found no significant relationship after metabolic covariate adjustment. This inconsistency likely reflects both the species-specific biology of resistin and the difficulty of measuring meaningful resistin levels in human populations where monocyte-derived, rather than adipocyte-derived, resistin predominates. Future research should prioritize human adipose tissue and immune cell studies that directly measure resistin’s effects on SASP factor secretion in the context of clinical obesity, alongside prospective cohort data linking circulating resistin to cancer incidence and SASP biomarker profiles. Effects of Resistin on IL-1β is illustrated in Fig. 5B.

A growing body of clinical evidence demonstrates that the SASP cytokines reviewed herein — IL-6, IL-1β, IL-8, and MCP-1 — are not merely mechanistic constructs derived from preclinical models, but measurable biomarkers in human clinical trials enrolling obesity-related cancer populations. Table 1 summarizes registered clinical trials in which one or more of these SASP factors were prospectively measured as primary or secondary outcomes in cancer patients or individuals at elevated cancer risk due to obesity. These trials span breast cancer, endometrial cancer, prostate cancer, and solid tumor survivors, and encompass a range of interventional designs including exercise, dietary weight loss, bariatric surgery, and pharmacological IL-1β blockade. Collectively, they validate the translational relevance of the obesity-SASP-cancer axis and highlight the emerging use of SASP cytokines as pharmacodynamic endpoints in obesity-cancer clinical research. Table 1 assesses SASP Cytokines in Clinical Trials in Obesity-Related Cancer Populations.

**Table 1:** Assessment of SASP cytokines in clinical trials in obesity-related cancer populations

NCT number	Cytokine(s) measured	Population	Trial title	Trial description
NCT02466061	IL-1β IL-6 IL-8	Endometrial Cancer Survivors (Obese/Overweight)	Transdisciplinary Research on Energetics and Cancer — Obesity and Weight Loss in Endometrial Cancer Survivors: A Randomized, Multi-site Trial (Lifestyle Beyond Cancer Study)	Multi-site RCT of weight loss intervention (diet + exercise) vs. attention control in obese endometrial cancer survivors. IL-6, IL-1β, and IL-8 measured as secondary biomarker outcomes to assess whether weight loss-driven SASP reduction correlates with improved cancer prognosis.
NCT02453139	IL-6	Obese and Non-obese Men with Advanced Prostate Cancer	Evasion of Immune Editing by Circulating Tumour Cells in an Exercise Modifiable Mechanism Underlying Aggressive Behaviour in Obese Men With Prostate Cancer	Observational study examining exercise effects on circulating tumour cell immune evasion in obese vs. non-obese men with advanced prostate cancer. Serum IL-6 measured as a primary obesity-cancer inflammatory mediator linking adiposity to aggressive tumour behaviour.
NCT02135562	IL-6	Obese Endometrial Cancer Survivors	A Pilot Study of a Protein-Sparing Modified Fast for Weight Loss in Obese Endometrial Cancer Survivors	Pilot study of protein-sparing modified fast (PSMF) diet in obese endometrial cancer survivors. Serum IL-6 measured as a biomarker of adipose tissue-driven systemic inflammation; reduction in IL-6 is assessed as a potential indicator of reduced cancer recurrence risk following weight loss.
NCT02152462	IL-6	Breast Cancer (Black Women — Obesity-Associated Biomarkers)	Exergaming Intervention and Breast Cancer Biomarkers in Black Women	RCT examining the effect of an exergaming (active video game) exercise intervention on breast cancer biomarkers in Black women. Serum IL-6 measured as an obesity-related inflammatory biomarker; trial addresses racial disparities in obesity-driven breast cancer inflammation.
NCT02431676	IL-6 IL-8	Cancer Survivors Previously Diagnosed with a Malignant Solid Tumor	Trial of Behavioral Weight Loss and Metformin Treatment to Lower Insulin Growth Factor in Cancer Survivors	RCT of behavioral weight loss plus metformin vs. placebo in cancer survivors. IL-6 and IL-8 measured as secondary SASP biomarker outcomes to assess whether combined weight reduction and metformin treatment reduces obesity-driven pro-inflammatory cytokine levels in cancer survivors.
NCT06046755	IL-6R	Obesity-Related Breast Cancer (During Active Oncological Treatment)	Evaluation of Changes in the Methylome and Prognosis of Obesity-related Breast Cancer After Nutritional Intervention-induced Weight Loss During the Oncological Treatment (NUTOBREST)	RCT of ketogenic vs. standard hypocaloric diet during oncological treatment of obesity-related breast cancer. IL-6 receptor (IL-6R) measured to assess the impact of weight loss on IL-6/STAT3 signaling; epigenetic (methylome) changes are the primary outcome, with IL-6R as a mechanistic SASP biomarker.
NCT03091842	IL-6 IL-8	Obese Breast Cancer Survivors	Targeting Adiposity and Inflammation With Movement to Improve Prognosis in Breast Cancer Survivors (AIM Trial)	Phase II RCT of circuit aerobic + resistance exercise vs. traditional exercise vs. attention control in 276 obese breast cancer survivors (Dana-Farber Cancer Institute). Serum IL-6 and IL-8 are pre-specified secondary outcome measures alongside TNF-α, hs-CRP, leptin, and adiponectin. Primary outcome: adipose tissue macrophage phenotype by core biopsy. (Source: Kang et al., Front Oncol 2022; PMID: 35814430)
NCT04540263	IL-1β IL-6 IL-8 MCP-1	Obese Patients at Elevated Cancer Risk (Bariatric Surgery Cohort)	Evaluation of Blood Cytokine Levels of Patients Who Underwent Sleeve Gastrectomy and Gastric Bypass Operations Due to Obesity	Observational study measuring 13 cytokines — including IL-1β, IL-6, IL-8, and MCP-1 (CCL2) — by flow cytometry before and after sleeve gastrectomy or gastric bypass in obese patients. Directly assesses whether surgical weight loss reduces the obesity-driven SASP cytokine panel associated with elevated cancer risk. (Source: ClinicalTrials.gov diagnostic test description)
NCT01327846 (CANTOS)	IL-1β	Obese/Metabolic Syndrome Patients — Pre-specified Lung Cancer Subanalysis	Antiinflammatory Therapy with Canakinumab for Atherosclerotic Disease (CANTOS Trial)	Landmark Phase 3 RCT of canakinumab (anti-IL-1β mAb) vs. placebo in 10,061 patients with prior MI and elevated hs-CRP, an obesity/metabolic syndrome-enriched population. Pre-specified exploratory analysis: canakinumab 150 mg q3 months produced significant dose-dependent reduction in lung cancer incidence (HR 0.33, *P* = 0.0002) and cancer mortality. Strongest human clinical evidence that IL-1β blockade in an obese/inflamed cohort reduces cancer incidence. (Source: Ridker et al., Lancet 2017; PMID: 28869257)

Abbreviations *RCT* Randomized Controlled Trial, *SASP* Senescence-Associated Secretory Phenotype, *TME* Tumor Microenvironment, *hs-CRP* high-sensitivity C-reactive protein, *IL* Interleukin, *MCP-1* Monocyte Chemoattractant Protein-1

NCT numbers verified against ClinicalTrials.gov registry

Cytokine measurements confirmed from registered outcome measures, published trial protocols, or ClinicalTrials.gov diagnostic test descriptions

## Conclusion

The evidence reviewed herein underscores that obesity is not merely a comorbid condition but a central driver of oncogenesis, primarily through the creation of a chronic inflammatory and immunosuppressive state. A pivotal, and increasingly recognized, mechanism is the obesity-induced acceleration of cellular senescence and the resultant SASP. The metabolic milieu of obesity, characterized by a distinct adipokine profile and insulin resistance, actively fuels the SASP, creating a feed-forward loop of inflammation that permeates the tumor microenvironment. This cascade, mediated by factors like IL-6, IL-8, MCP-1, and IL-1β, promotes hallmark capabilities of cancer cells while simultaneously erecting barriers to effective anti-tumor immunity and conventional therapies.

The interplay between metabolic hormones — leptin, adiponectin, resistin, and insulin — and the SASP cytokine network provides a molecular framework that may explains the heightened cancer risk and worsened prognosis observed in individuals with obesity. This integrated framework reveals multiple therapeutic vulnerabilities at distinct points in the obesity-SASP-cancer cascade. Pharmacological senescent cell clearance with senolytic agents (dasatinib, quercetin, navitoclax) has demonstrated proof-of-concept feasibility in early human studies and is now entering early-phase oncology trials. IL-1β blockade has yielded the most compelling clinical signal to date, with the CANTOS trial demonstrating that canakinumab reduces incident lung cancer and cancer mortality in a metabolically inflamed population, a finding that directly validates the mechanistic axis reviewed herein, even as its broader oncological application awaits prospective confirmation. Metabolic interventions including metformin offer a clinically immediate and accessible route to reducing SASP-driving hyperinsulinemia, inflammasome activation, and adipose tissue dysfunction, though definitive prospective trial evidence for oncological benefit remains limited and mixed. A critical unresolved challenge across all three therapeutic strategies is the absence of validated, obesity-specific SASP biomarkers distinguish their obesity-driven component from age-related senescence, and predict therapeutic response. Developing such biomarkers, in parallel with adipokine profiling panels (leptin/adiponectin ratio, resistin, circulating IL-6, and IL-1β), represents a critical translational priority. Ultimately, the most effective clinical approach will likely require combination strategies that simultaneously address adipose tissue dysfunction (metabolic interventions and weight management), SASP amplification (senolytics or senomorphics), and specific pro-tumorigenic cytokines (IL-1β or IL-6 blockade), integrated within personalized oncology frameworks that stratify patients by metabolic phenotype. Advancing this agenda requires purposefully designed clinical trials that embed mechanistic biomarker assessment, enroll patients stratified by obesity-associated metabolic dysregulation, and use SASP cytokine dynamics as pharmacodynamic endpoints — a research priority that the mechanistic insights of this review are intended to support.

### Future Directions and Clinical Implications

Several translational priorities emerge from the mechanistic gaps identified in this review.

First, the development of validated, obesity-specific SASP biomarker panels that reliably distinguish obesity-driven senescence from age-related senescence remains the most critical unresolved challenge in the field; without such tools, patient stratification for SASP-targeted trials is not feasible.

Second, future clinical trials in obesity-related cancers should embed SASP cytokines, specifically IL-6, IL-1β, IL-8, and MCP-1, as pre-specified pharmacodynamic endpoints rather than secondary exploratory measures, enabling direct mechanistic validation of the obesity-SASP-cancer axis in human populations.

Third, combination strategies that simultaneously target adipose tissue dysfunction through metabolic intervention, SASP amplification through senolytic or senomorphic agents, and specific pro-tumorigenic cytokines through targeted blockade have not been tested in any clinical trial and represent the logical next step beyond the single-agent approaches evaluated to date. Advancing this agenda requires trials that enroll patients stratified by metabolic phenotype including BMI, adipokine profile, and degree of insulin resistance rather than treating obesity as a binary covariate.

### Limitations

Several important limitations apply to the mechanistic framework presented in this review.

First, the majority of mechanistic evidence linking obesity-associated adipokines to SASP amplification and pro-tumorigenic cytokine production derives from in vitro cell line experiments and murine dietary obesity models with little clinical trials (mentioned in Table 1). While these models have provided foundational mechanistic insight, they may not fully replicate the complexity and heterogeneity of human obesity-associated malignancies.

Second, the attribution of cancer risk to adipose-driven immunopathology specifically remains difficult to disentangle in human observational data. Establishing causality and identifying the dominant mechanistic pathway in clinical populations are central challenges in the field. The epidemiological association between obesity and cancer risk is consistently demonstrated, but the mechanistic primacy of SASP-mediated immune remodeling over other obesity-associated oncogenic pathways has not been established in prospective human studies.

Third, the cancer-preventive effects attributed to adiponectin in epidemiological studies may be confounded by correlated variables including insulin sensitivity, physical activity, and overall metabolic health — all of which track with adiponectin levels and independently affect cancer risk. Whether the inverse associations between adiponectin and cancer incidence are causally driven by adiponectin itself or by these correlated metabolic parameters cannot be determined from observational data. The adiponectin-IL-8 and adiponectin-MCP-1 evidence bases are more limited and predominantly mechanistic, without convergent human epidemiological support.

Finally, a critical limitation across the entire mechanistic framework reviewed is the absence of validated, obesity-specific SASP biomarkers that reliably distinguish the obesity-driven component of SASP from age-related senescence in human clinical populations. Without such biomarkers, it is not possible to confirm in prospective clinical settings whether the adipokine-SASP-cancer axis operates as the mechanistic framework presented in this review proposes, or to identify which patients with obesity are most likely to benefit from SASP-targeted therapeutic interventions. Developing and validating such biomarkers, in parallel with adipokine profiling panels, represents the most critical translational priority identified by this review. 

## Data Availability

No datasets were generated or analysed during the current study.

## Key References

- Jin X, Qiu T, Li L, et al. Pathophysiology of obesity and its associated diseases. Acta pharmaceutica Sinica B. 2023;13(6):2403–2424.
  - ○ Links between obesity and cancer.
- Burgos-Molina AM, Téllez Santana T, Redondo M, et al. The Crucial Role of Inflammation and the Immune System in Colorectal Cancer Carcinogenesis: A Comprehensive Perspective. 2024;25(11):6188.
  - ○ Release of proinflammatory cytokines (IL-6; IL-17; TNF-α) foster tumor proliferation, survival, and invasion.
- Caronni N, La Terza F, Vittoria FM, et al. IL-1β+ macrophages fuel pathogenic inflammation in pancreatic cancer. Nature. 2023;623(7986):415–422.
  - ○ Targeting IL-1β may enable preventive or therapeutic strategies for reprogramming immune dynamics in pancreatic cancer.
- García-Miranda A, Montes-Alvarado JB, Sarmiento-Salinas FL, et al. Regulation of mitochondrial metabolism by autophagy supports leptin-induced cell migration. Scientific Reports. 2024;14(1):1408.
  - ○ Leptin-induced mitochondrial metabolism promoted cancer cell migration.
- Chen YL, Ma YC, Tang J, et al. Physical exercise attenuates age-related muscle atrophy and exhibits anti-ageing effects via the adiponectin receptor 1 signalling. Journal of cachexia, sarcopenia and muscle. 2023;14(4):1789–1801.
  - ○ AdipoR1 pathway has an impact on the anti-ageing benefits of exercise

## Declarations

### Figure Permissions

All figures were created using BioRender (BioRender.com) under an authorized institutional license. Figures are original compositions created by the author(s) and are published with permission from BioRender in accordance with the terms of the license agreement.
